# An experimental model to induce digital dermatitis in beef calves

**DOI:** 10.1186/s12917-022-03345-x

**Published:** 2022-06-23

**Authors:** Anice D. Thomas, Edmond A. Pajor, Benjamin Caddey, Christy Goldhawk, Larissa Martins, Karin Orsel

**Affiliations:** Department of Production Animal Health, Faculty of Veterinary Medicine, University of Calgary, 3280 Hospital Drive NW, Calgary, AB T2N 4Z6 Canada

**Keywords:** Hairy heel warts, Cattle, Experimental infection, Induction model, Behaviour, Pain

## Abstract

**Background:**

Digital dermatitis (DD) is a multifactorial infectious disease affecting the skin on feet of cattle causing erosion and inflammation above the heel bulbs. Some cases of DD cause lameness and significantly impact animal welfare and productivity. While DD has emerged as a concern for the beef industry, key information regarding early detection and its impact on cattle behaviour is lacking. The primary objective of this study was to determine if an established DD experimental model for dairy calves could be used to induce DD lesions in beef calves. A secondary objective was to describe changes in behaviour and pain associated with induction of DD lesions. Eight beef calves acquired from a single cow-calf operator were enrolled in the study. Upon enrolment, calves were evaluated and determined to be free of foot lesions. Within the experimental environment, calves were housed in individual pens and assigned to two groups (mock-inoculated and inoculated). Both hind feet of each calf were enrolled. Within calf, inoculation protocol was consistent, and a 28-day experimental protocol was employed. Two days prior to inoculation, both hind feet of each calf were abraded (area above the heel bulbs and below the dewclaws), moistened, and wrapped to facilitate an anaerobic condition. Feet were inoculated with macerated DD lesion material or mock inoculum and remained wrapped until clinical signs of DD or protocol endpoint.

**Results:**

After a period of 14 to 18 days post inoculation, three of five inoculated calves developed clinical signs (lameness), and upon close inspection, DD lesions were present on at least one hind foot. Two of five inoculated calves did not develop lesions within 28 days. Zero of three mock-inoculated calves developed DD. *Treponema* spp. were detected by quantitative polymerase chain reaction from biopsies of induced lesions. Measurements of behaviour prior to disease induction were numerically different between DD affected and mock-inoculated calves.

**Conclusions:**

An experimental infection model established for dairy cattle was used to successfully induce acute DD lesions in three of five inoculated beef calves. This model can provide a framework to study intervention protocols and to evaluate the impact of DD on behaviour and pain.

## Background

For nearly half a century, digital dermatitis (DD), an infectious skin disease affecting the feet of cattle, has plagued the dairy industry worldwide [1], and has been the focus of considerable research. In contrast, only a few geographic regions: the United Kingdom [2], Australia [3] and North America [4–6], have reported the disease in beef cattle. The impact of DD in beef cattle is not well understood as parallels with dairy cattle might not always be valid [7]. For both dairy and beef cattle, some cases of DD present with lameness, which significantly impacts productivity and animal welfare, while other cases do not [8–10].

Digital dermatitis is commonly characterized by ulcerative and necrotic lesions in the pastern area between the heels bulbs [11, 12] which initially presents as small (< 2 cm), circumscribed, red to grey epithelial defects with edges forming a white margin [13]. Several classification systems are available in the literature to describe and score the development of DD macroscopically [14–19]. The M-stage scoring system (‘M′ for Mortellaro), a 6-point DD classification system [14, 15] is the most widely used. Irrespective of the scoring system employed, detection of cattle with DD at disease onset is difficult within commercial beef production systems. Early detection is key for treatment success as cure rates increase when DD is identified early in the disease cycle [20], lessening the animal welfare impact. Unlike dairy cattle, beef cattle are infrequently handled and primarily observed for foot lesions and other health complications during daily pen checks. In a manner most comparable to what is practiced in beef cattle, Jacobs et al. [21] observed dairy young stock in their pens for the presence or absence of DD and compared those results to DD scores obtained when their feet were lifted and visually appraised in a trimming chute (‘Reference Test’). Using the M-stage scoring system they reported sensitivity and specificity of 23% and 83% respectively for identifying early-stage lesions (M1) at the foot level, demonstrating the difficulty with early detection. Wet and muddy pens also increase the difficulty of identifying early-stage lesions during pen checks.

It is generally accepted that DD is a polymicrobial, multifactorial infectious disease with the primary causative agents belonging to the bacterial group spirochetes, specifically *Treponema* spp. [22–24]. The exact aetiology of DD in dairy cattle is still unclear and attempts to induce DD in an infection model from pure cultures have largely failed [25, 26]. There are two published reports of successful protocols utilizing DD lesion homogenate to induce DD in dairy cattle. After an extensive wrapping procedure and double inoculation with fresh DD biopsy homogenate, Gomez et al. [25] achieved DD induction in yearling Holstein heifers. Krull et al. [26] also used fresh DD lesion homogenate to induce DD in Holstein calves and achieved a 95% induction success over a 28-day protocol having utilized a single inoculation procedure. Successful induction of DD has also been reported in sheep. Wilson-Welder et al. [27] inoculated crossbred sheep with bovine derived DD inoculum and achieved an 88% induction over a 28-day protocol, and contagious ovine digital dermatitis was induced in 15 of 30 experimental sheep [28]. Most recently, Wilson-Welder et al. [29] using lesion material obtained from wild elk induced treponeme-associated hoof disease in sheep by way of a sheep DD model. To date there have been no published reports that have attempted to experimentally induce DD in beef cattle.

Investigating DD treatment interventions and diagnostics within a commercial beef production setting is challenging as cattle with early-stage lesions are difficult to identify and external factors such as management practices, pen environment and infection pressure confound disease development. An experimental model allows for control of potential confounders (measured and unmeasured), namely animal selection, disease exposure time and concentration, and the physical environment [30]. Experimental disease models are excellent for determining causation and developing intervention protocols. The primary objective of this study was to determine if an established DD experimental model for dairy calves can be used to induce acute DD lesions in beef calves. A secondary objective was to describe changes in behaviour and pain associated with induction of acute DD lesions.

## Results

### Inoculation site preparation

Foot wrap integrity varied throughout the experiment and all eight calves, mock-inoculated (MI, *n* = 3) and inoculated (IN, *n* = 5), required periodic wrap reinforcement. Prior to and on inoculation day, five calves, from both groups (MI = 1; IN = 4) lost their foot wrap completely from one or both hind feet and required a new foot wrap. Again, on day 18, two calves in the IN group, lost their foot wrap completely on one or both hind feet which had to be rewrapped.

### Experimental infection and clinical evaluation

**Fig. 1:**
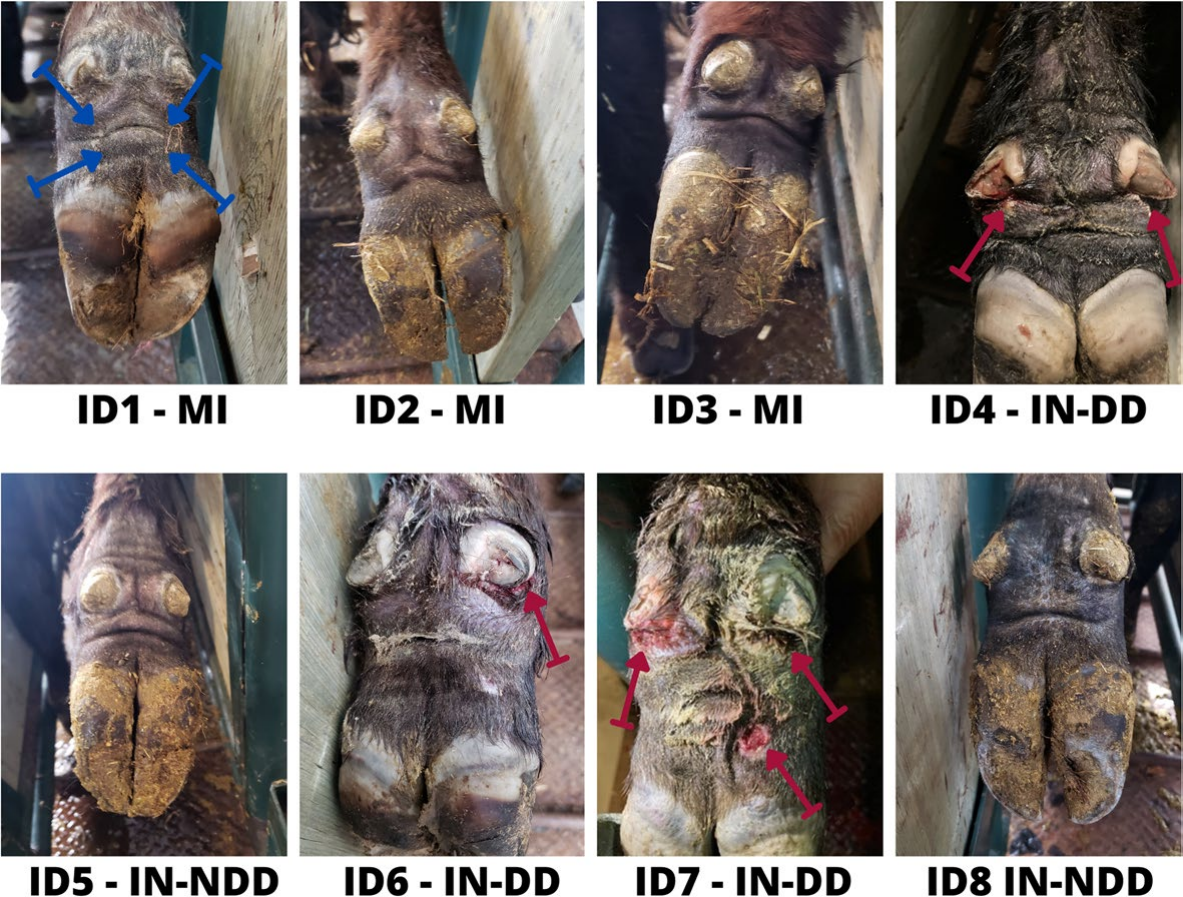
Representative lesions from digital dermatitis (DD) experimental model. ID = calf identification; MI = mock-inoculated calves (no DD lesions); IN-NDD = inoculated calves (no DD lesions); IN-DD = inoculated calves with a clinical case of DD (red arrows); Blue arrows = location of abrasion sites (four regions: two above the heel bulbs, above the medial and lateral claws, and two below the dewclaws on each side of the pastern area. Colour version available online

Three of five IN calves were confirmed with a clinical case of DD (IN-DD); Table 1. Calves were diagnosed with DD on day 14 and day 18 of the experiment after presenting lameness. Lesions were observed above the heel bulbs (HB) and below the dewclaws (DC); Fig. 1; red arrows. Lesions presented as small, moist, circumscribed ulcers consistent with early-stage (M1) DD lesions [14, 15]. Pressure sores and dermatitis were observed in the DC area when foot wraps were removed.

**Table 1:** Clinical outcome of the experimental induction of beef calves with digital dermatitis (DD)

Experimental Group^a^	Identification	Left Hind^b^	Right Hind^b^	Lesion Prevalence^c^
MI	ID1	NDD	NDD	0/2
MI	ID2	NDD	NDD	0/2
MI	ID3	NDD	NDD	0/2
IN	ID4	DD	DD	2/2
IN	ID5	NDD	NDD	0/2
IN	ID6	DD	NDD	1/2
IN	ID7	DD	NDD	1/2
IN	ID8	NDD	NDD	0/2

^a^*MI* mock-inoculated (no DD lesion material), *IN* Inoculated (inoculated with DD lesion material)

^b^*NDD* no DD lesion present, *DD* clinical case of DD, based on Döpfer et al., [14]

^c^Number of feet with lesions/number of feet examined

Two of five IN calves did not present with DD (IN-NDD) in either hind foot by the end of the experiment. Both calves lost their foot wrap completely from one or both hind feet within 2 weeks of being inoculated and required a new foot wrap. At experimental endpoint all abraded areas were scabbed over or healed, no obvious skin break. Swelling was observed around the DC.

None of the hind feet of the three MI calves presented with DD by the end of the experiment. Further, all abraded areas had completely healed and appeared normal when examined at the experimental endpoint.

Lameness was observed in all IN-DD calves. Lameness presented as early as day 11 and persisted until foot wrap removal and treatment of lesions. One calf in the MI group presented with lameness on day 25 of the experiment. Clinical inspection of the lame hind foot determined DD was not present, and lameness was associated with the foot wrap. Lameness lessened once the foot wrap was removed.

### Quantitative real-time polymerase chain reaction (qPCR)

**Fig. 2:**
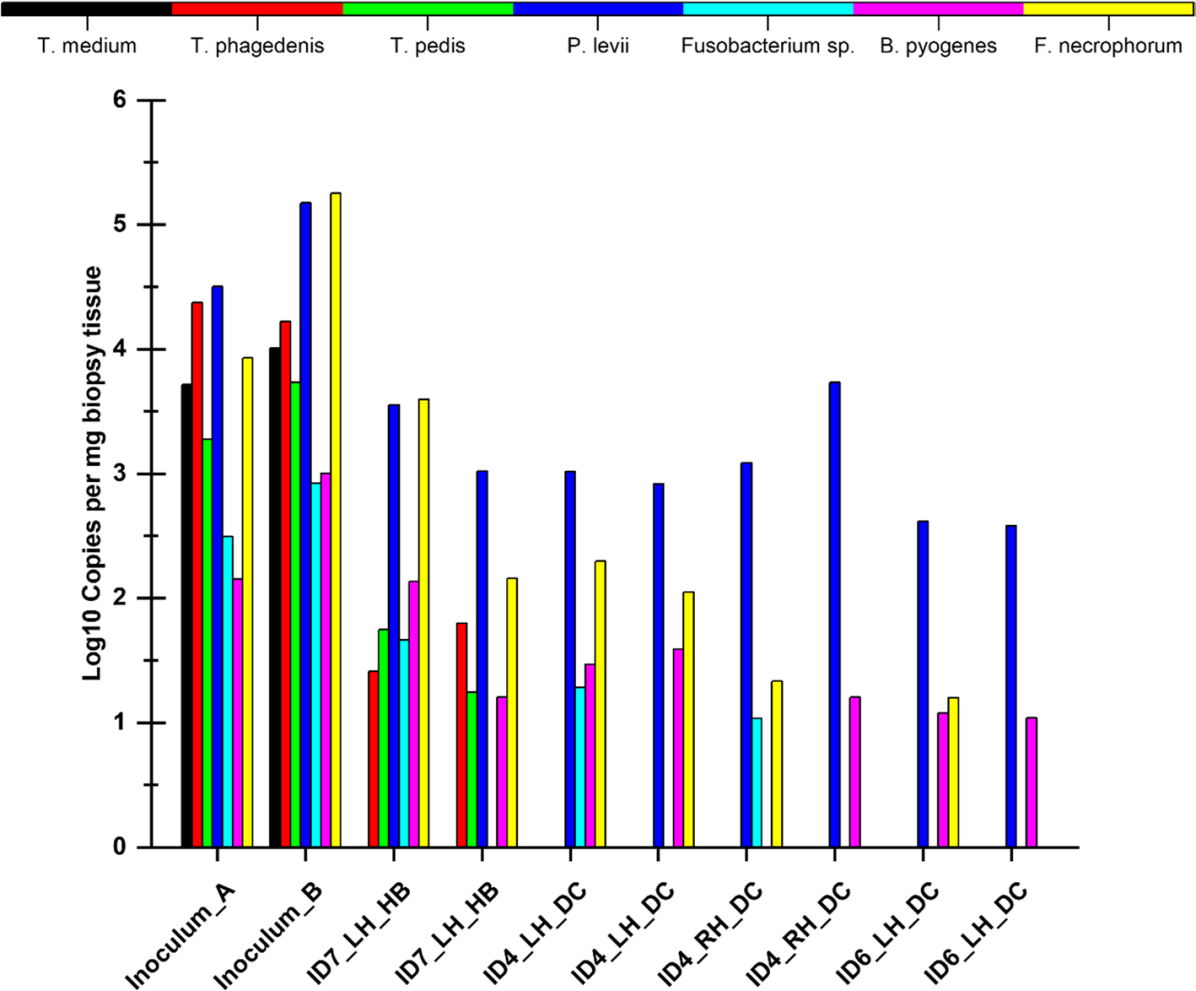
Absolute abundance of each bacterial species isolated from the inoculum and biopsies taken from experimentally induced DD calves. Species copy numbers were standardized by the weight of the biopsy tissue. A pseudocount of 1 was added before log transforming bacterial copy numbers. Inoculum_A = inoculum at the start of the induction protocol; Inoculum_B = inoculum at the end of the induction protocol; ID = calf identification; LH = left hind foot; RH = right hind foot; HE = heel bulbs; DC = dewclaws. Colour version available online

In total eight biopsies were taken from the IN-DD calves. Calf identification (ID), foot biopsied (left hind or right hind), lesion location (DC or HB) and anaerobic bacteria detected from biopsies are reported in Fig. 2. The bacterial species detected from biopsies included *Treponema phagedenis, Treponema pedis, Porphyromonas levii, Fusobacterium sp., Bacteroides pyogenes and Fusobacterium necrophorum*. *T. medium* was undetectable in all biopsy samples. *Treponema* spp. was identified in only two biopsy samples. *P. levii* was identified with the highest abundance in all biopsy samples. Apart from *T. medium* all other bacterial species identified in the inoculum were identified in at least one IN-DD biopsy sample (Fig. 2) and no other bacteria species were identified in biopsy samples than what was detected the inoculum.

### Behaviour and pain

**Fig. 3:**
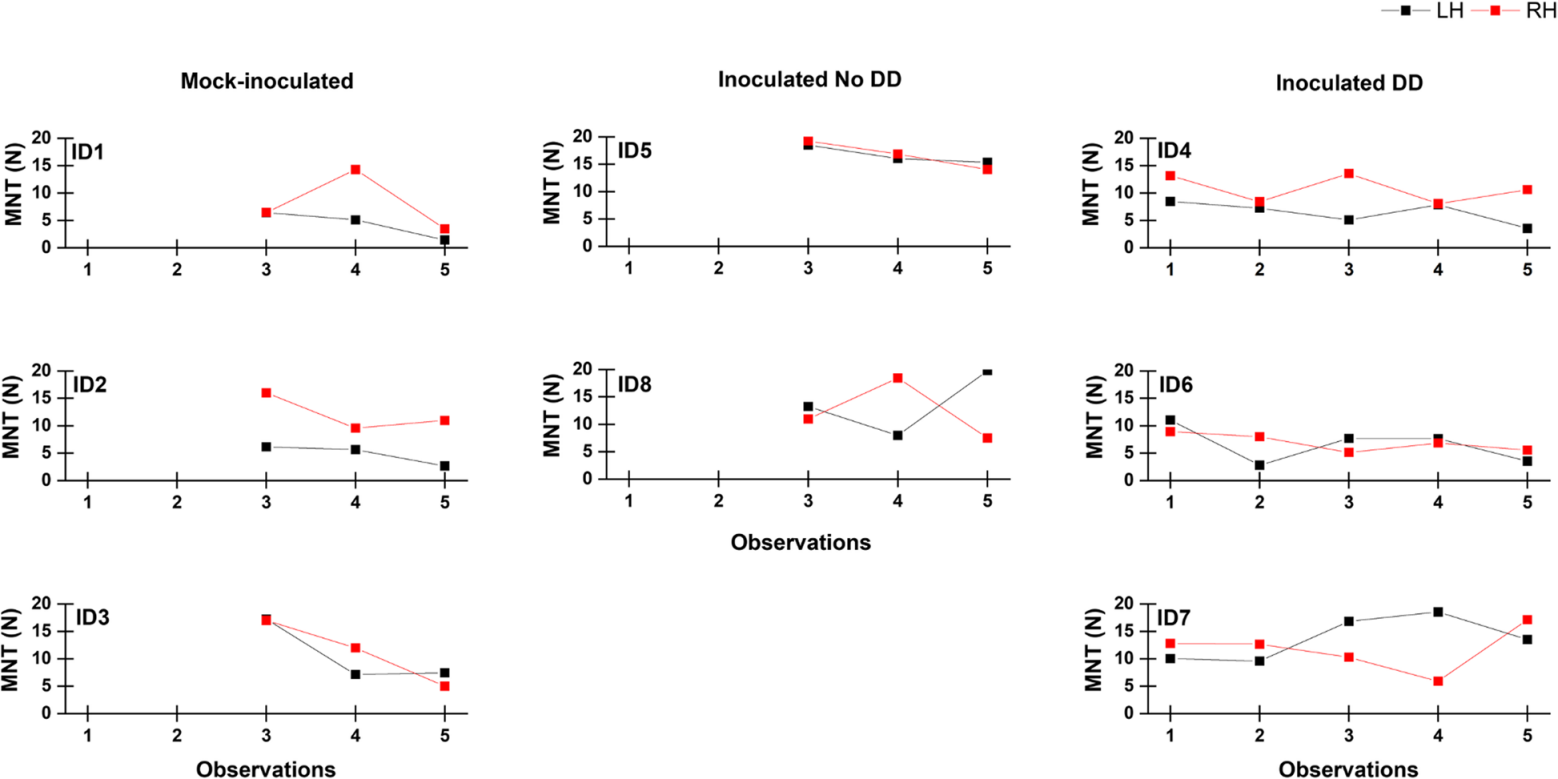
Mechanical nociceptive threshold (MNT) in hind feet of beef cattle experimentally induced with digital dermatitis (DD). MNT = mechanical nociceptive threshold in Newtons (N); ID = calf identification; Mock-inoculated = no DD lesions; Inoculated No DD = no DD lesions; Inoculated DD = calves with a clinical case of DD; Observations = observation number where 1 is acute DD lesion at diagnosis, 3 is healing lesion (IN-DD) or abrasion site (MI; IN-NDD), and 5 is healed lesion (no sign of pre-existing lesion) or healed abrasion (no sign of abrasion site); LH = left hind foot; RH = right hind foot. Colour version available online

Daily mean percent time spent ruminating, feeding, active, inactive, and standing was similar between groups and presented in Table 2. Mechanical nociceptive threshold (MNT) was measured for calves in the IN-DD group. There were five observation periods, from DD diagnosis (acute lesion) to healed lesion (no sign of pre-existing lesion). Within animal there were no trends in MNT over the observation periods. Average MNT by hind foot by observation number is reported in Fig. 3. Calves in the MI and IN-NDD group kept their foot wraps on until the end of the 28-day protocol whereas calves in the IN-DD group had their foot wraps removed prior to day 28. Consequently, calves in the IN-DD group have two pain measurements (observations 1 and 2) taken prior to day 28.

**Table 2:** Daily behaviour patterns of beef calves experimentally induced with digital dermatitis (DD)

Behaviour^a^	Group^b^	N	Mean	SE	95% CI	
Rumination Time (%)	MI	3	25.8	2.7	19.8	31.9
	IN-NDD	2	30.1	3.3	22.7	37.6
	IN-DD	3	35.0	3.9	26.4	43.6
Feeding Time (%)	MI	3	20.3	1.8	16.2	24.4
	IN-NDD	2	22.3	2.2	17.3	27.3
	IN-DD	3	18.8	2.6	13.0	24.6
Inactivity Time (%)	MI	3	29.2	1.1	26.7	31.6
	IN-NDD	2	29.7	1.3	26.7	32.8
	IN-DD	3	29.4	1.6	25.9	32.8
Activity Time (%)	MI	3	24.6	3.2	17.5	31.8
	IN-NDD	2	17.8	4.0	9.0	26.6
	IN-DD	3	16.8	4.6	6.6	27.0
Standing Time (%)	MI	3	32.1	3.7	23.6	40.6
	IN-NDD	2	32.2	5.3	20.2	44.2
	IN-DD	3	29.1	5.3	17.1	41.1

^a^Mean percent time spent daily per behaviour 5 to 1 day before DD diagnosis

^b^*MI* Mock-inoculated (no DD lesion), *IN-NDD* Inoculated not diseased (no DD lesion), *IN-DD* Inoculated diseased (clinical case of DD, M1); based on Döpfer et al., [14]

## Discussion

The primary aim of this study was to determine if an effective protocol, previously used to induce DD in Holstein calves, could also induce DD in beef calves. Using the previously validated dairy protocol [26], acute DD lesions were experimentally induced, consistent with abrasion locations. No MI calf was diagnosed with DD. These calves were housed in a separate area of the barn, away from the IN group, and were always processed first in the chute to minimize the potential for cross contamination. Additionally, all equipment such as feeding containers, abrasion tool, ropes and syringes were assigned individually to calves. Because MI and IN calves were housed separately blinding was not possible at group allocation, inoculation, assessment, and data analysis.

Based on the same induction protocol Krull et al. [26] observed 95% induction rate at the foot-level, for naïve dairy calves exposed to fresh macerated DD inoculum in all four feet. In our study, we used two feet and animal-level induction rate was 60%. Animal-level induction rate is the best way to determine induction rate for our study, as calves were treated and removed from the study once DD was induced in at least one hind foot. Lower induction rate in our study may be explained by the loss of foot wrap integrity throughout the study for IN-NDD calves. Both calves in the IN-NDD group completely lost their foot wrap from one or both hind feet within 2-weeks of being inoculated. At time of re-wrapping, skin of the feet were dry, and with the wrap completely off, the foot no longer met the condition of being in a moist anaerobic environment, which has been shown to be necessary for DD induction [25, 26], nor were feet re-inoculated. Induction rate may also have been affected by skin thickness and the depth of abrasion. In our study we inoculated weaned calves that were 6 to 9 months older than the calves in Krull et al. [26], and skin thickness has been shown to increase with age [31]. Nevertheless, the fact that we were able to reproduce DD lesions confirms that this model is suitable for use in beef calves.

Bacteria detected in biopsied tissues in our study were consistent with the inoculum used. Biopsy preparation could have influenced bacteria present in the biopsies, knowing this we tested a sample of the inoculum at the start of the induction protocol and at the end of the induction protocol. Based on the limited difference we observed between both samples we believe there was little to no impact of culture or bacteria survival rate, however there could have been a difference in bacteria survival times. We had a relatively large proportion of host DNA in our biopsy DNA extractions which could impact our qPCR results since host DNA may inhibit the PCR reaction. We diluted our DNA templates to try and limit this issue. *Treponema* spp. were not detectable in all biopsy tissues, even though identified in the inoculum. In addition, *T. medium* although identified in the inoculum material used in our study, was not detectable in any of the biopsy tissues. These findings could be because *T. medium* quantities are outside of the range of the qPCR assays used in our study, or the role of *T. medium* in development of DD is minimal; further studies would be recommended to validate its association with disease onset. Overall, there are several *Treponema* spp. that are identifiable in DD lesions [19, 32, 33], but currently existing DD-validated *Treponema* qPCR assays are limited to only the three *Treponema* spp. analysed in our study, therefore, further studies are warranted to characterize *Treponema* spp. distribution in early-stage DD lesions. *P. levii* was detectable in all biopsy samples and warrants further investigation into its association with disease onset. Caddey et al. [22] reported that early-stage DD lesions (M1) were associated with *P. levii,* which was detectable in 100% of the M1 lesion samples biopsied. Unlike in field studies, where DD lesions and their bacterial composition are studied throughout disease development our study identified DD at the beginning of the disease cycle and the corresponding bacteria could reflect the bacterial species present at disease onset. On day 28 all existing wraps on the MI and IN-NDD groups were removed. Within both these groups there were no DD lesions present and the skin was intact. No biopsies were therefore taken as we know from our previous work the bacterial composition of non-DD biopsies [22]. Additionally, we did not want to compromise calf welfare any further by inflicting more pain and stress. As our sample size was small and we did not take biopsies from feet of calves in the MI or IN-NDD groups we were unable to further compare or explore the bacterial composition in our DD biopsy samples. Further studies characterizing the microbiota of early-stage DD lesions is necessary to understand how these pathogens and others contribute to disease onset; crucial information to inform early disease intervention strategies.

Maintaining foot wrap integrity throughout the experiment was a major challenge. Prolonged moisture and an anaerobic environment were not maintained for the duration of the experiment. These conditions are essential for DD induction in experimental models as demonstrated by Gomez et al. [25], Krull et al. [26], and Wilson-Welder et al. [27]. Additionally, the most common risk factors identified for the natural development of DD are wet and muddy pen conditions [12, 34]. Further studies investigating the best wrapping method to achieve this condition is warranted especially when using bigger, more active calves. Compared to other DD experimental studies [25, 26], our wrapping method used fewer wrapping layers, was less invasive, and feet were wrapped vertically to allow for foot growth and blood flow in case of swelling. Additionally, when foot wraps fell off, the foot was observed to be dry indicating feet likely needed more frequent re-moistening to support microbial growth. Wrapping style also merits further investigation as pressure from the foot wrap on the DC area resulted in pressure sores. These sores in the DC area could have potentially increased susceptibility to infection in this location. Throughout the experiment, pen bedding was kept dry, thus pen environment did not contribute to foot wrap lost.

We used an ear accelerometer, video recordings, and an algometer to measure behaviour and pain. The ear accelerometer measured daily time spent ruminating, feeding, high activity, activity, and inactivity, and daily time spent standing were extracted from videos. Although numeric differences were observed, the small sample size and lack of power excludes statistical comparisons of behaviours prior to disease onset between groups. Additionally, differences in age and diet preclude comparison to other studies reporting behaviour of beef cattle as it has been shown that behaviours like rumination and feeding are sensitive to diet, dry matter intake, days on feed, body weight (BW), and stress [35, 36]. Pain measurements were taken over five periods for the IN-DD group. As with behaviour, numeric differences were observed however further statistical analysis was not possible due to sample size.

## Conclusions

Acute DD lesions were experimentally induced in beef calves using an established experimental protocol created for dairy calves. Behaviour traits: ruminating, feeding, activity, inactivity, and standing were measured using a non-intrusive equipment and did not interfere with the disease induction process. This protocol does not replicate the dynamics of DD on farm; however, it has the potential to be useful in future studies researching the impact of DD on beef cattle behaviour, pain and for testing intervention strategies.

## Methods

### Experimental animals

**Fig. 4:**
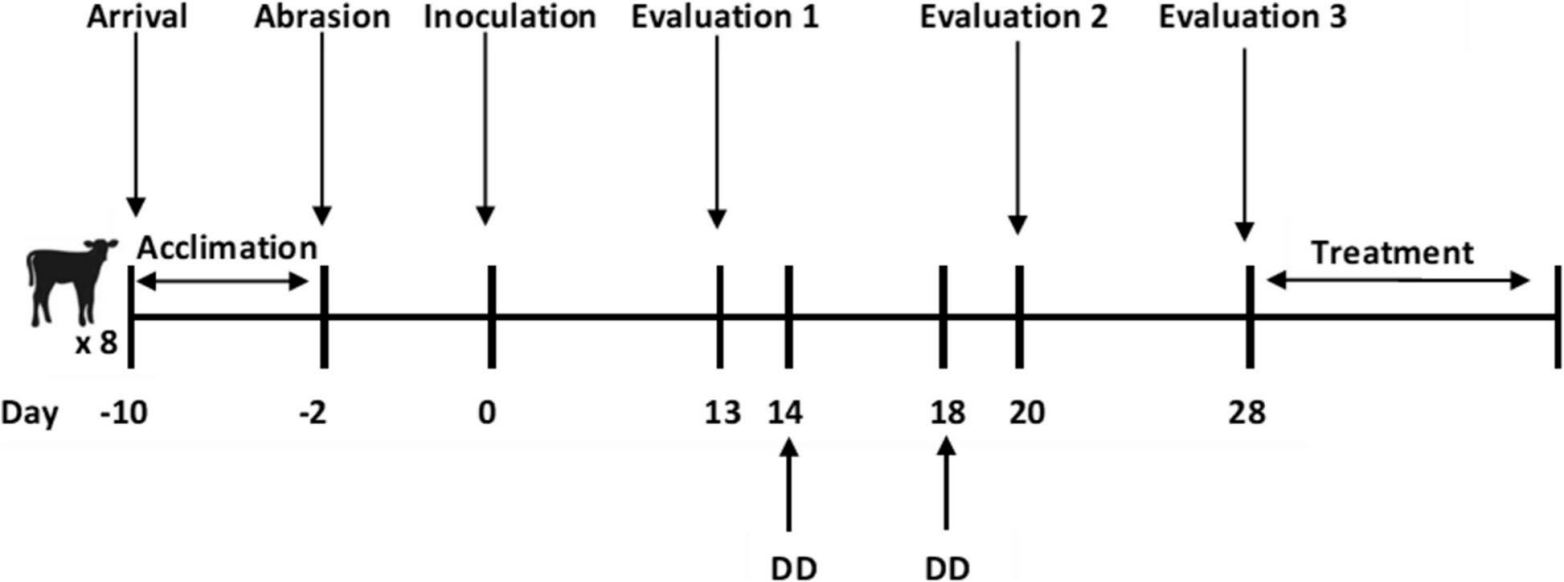
Timeline of the experimental induction of beef calves with digital dermatitis (DD). Periods of acclimatization, abrasion (including attachment of ear accelerometers), inoculation, evaluations 1 and 2 (re-moistening; foot warp reinforcement), evaluation 3 (foot wrap removal; clinical appraisal for DD; pain assessment; treatment), interval days between events, and when DD diagnosis was made

Beef calves, less than 1 year of age, with initial BW between 227 and 273 kg were sourced from a single cow-calf operation located in Alberta, Canada. For inclusion, study calves needed to be weaned, on feed (forage), tested negative for Bovine Viral Diarrhea Virus, and free from any clinical sign of foot disease. The “resource equation” was used to determine sample size [37, 38]. Eight calves were enrolled in the study to allow for group comparison with repeated measures, biological variation, and attrition losses. The study was conducted from January 2021 to March 2021 at the Veterinary Science Research Station (quarantine barn), University of Calgary, Canada. On arrival (day -10; Fig. 4), calves that were mixed during transport, were assigned to individual pens (10 m^2^) based on the order in which they entered the barn. The first three calves that entered the barn were assigned to the MI group and the remaining five assigned to the IN group. Pens had concrete floors, lined with rubber mats, bedded with straw, and were spot cleaned daily and cleaned in its entirety once per week. No pens shared the same sides. Pens had both full and partial side panelling allowing calves to see and hear each other but unable to touch. Calves were fed hay and calf grower twice per day and provided water ad libitum. Calves were observed daily for inappetence.

### Induction model

The induction model was in accordance with published methods to induce DD in dairy calves [26]. The study was designed to have a maximum duration of 28 days, preceded by an acclimatization period of eight days to allow calves to adapt to their new environment. Disease challenge was at the animal level and both hind feet within calf were allocated to the same protocol (MI or IN). Observations were made both at the animal and foot level.

#### Inoculum material

Digital dermatitis lesion material was used to create the inoculum. Skin biopsies from DD lesions were collected from donor beef heifers that were naturally infected with DD. The donor heifers were sourced from a single commercial feedlot located in Alberta, Canada. Six heifers, from two pens, presenting with a clinical case of DD in at least one hind foot were sampled. A clinical case of DD was defined using the M-stage scoring system [14, 15]. As per feedlot protocol for rehandling animals, the donor heifers were restrained in a squeeze chute and the affected hind foot lifted and restrained with a rope. The lesion was cleaned with a brush, rinsed with water, dried with paper towel, and max 2 ml lidocaine (Lidocaine HCl 2%, DIN 00,712,884, Zoetis Canada Inc., Kirkland, QC) administered subcutaneously per biopsy site. After 2 — 3 min, a biopsy punch (4 mm diameter, Integra Miltex, Kai medical, Dallas, TX) was used to core the outer superficial layer of the lesion. Biopsies were then placed in anaerobic transport medium (ATM; Anaerobe Systems, Morgan Hill, CA) and transported to the laboratory (University of Calgary, Canada) at room temperature. A total of 16 biopsies were taken and represented samples from heifers with M1 lesions (*n* = 2), M2 lesions (*n* = 9), and M4 lesions (*n* = 5); Döpfer et al. [14].

Within 2 h, DD biopsies arrived at the laboratory. In an anaerobic chamber (5% CO_2_, 5% H_2_, and 90% N_2_; Bactron3000, Sheldon Manufacturing Inc., Cornelius, Oregon, USA) biopsies were combined in a petri dish and macerated with a scalpel blade. The macerated lesion material along with 80 ml of growth media and 20 ml of Fetal Bovine Serum (Sigma-Aldrich, St. Louis, MO) were combined to make the inoculum. The growth media contained 40% MTGE (Anaerobic Systems, Morgan Hill, CA), 30% Brain Heart Infusion Broth (BD and Company, Sparks, MD), 15% Trypticase Arginine Serine Broth [39], and 15% Mueller Hinton Broth (BD and Company, Sparks, MD). The inoculum was then added to syringes which were secured in sterile Ziplock bags (2 per bag) prior to being removed from the anaerobic chamber for transportation. Each syringe contained 10 ml of inoculum. Four aliquots of the inoculum were taken to determine bacterial species present using qPCR. Two of the aliquots were stored at -80^O^C immediately after removal from the anaerobic chamber, and the other two were taken to the quarantine barn for the duration of the inoculation procedure, then returned to the laboratory and stored at -80^O^C to mimic the impact of transportation and environment on the inoculum. All four aliquots contained *T. phagedenis, T. medium, T. pedis, P. levii, Fusobacterium sp., B. pyogenes and F. necrophorum* as detected by qPCR [40]. Growth media, 10 ml per syringe were secured in sterile Ziplock bags (2 per bag) for use in the MI group.

#### Inoculation site preparation

Two days prior to inoculation (day -2; Fig. 4), cattle were moved to a manual squeeze chute and both hind feet were abraded. Each hind foot in turn, was lifted and tied with a rope above the DC to allow for safe access. An AGC 1-Speed Detachable Blade Clipper Kit (Andis, Sturtevant, WI) was used to clip the hair in the pastern area above the HB and below the DC. Four regions: two above the HB, above the medial and lateral claws, and two below the DC on each side of the pastern area (Fig. 1; blue arrows), were abraded with a Dremel Lite tool (Mount Prospect, IL), set at speed 2 using a Tungsten Carbide Cutter bit. The intent was to remove the epidermis without causing bleeding or disrupting the dermis (visually determined). Following abrasion, a 4 × 4 gauze pad was soaked with 10 ml of growth media and placed over the abraded area. A 12-inch tubing butterfly catheter (needle removed) was then placed on the gauze pad so that the tip was directly over the abraded area to allow for direct placement of DD inoculum onto the abraded sites. The catheter was secured to the foot with a strip of 2″ Gorilla Tape (Gorilla Glue, Inc., Sharonville, OH). The foot was then wrapped with a layer of brown cling gauze (Western Drug Distribution Center Limited, Edmonton, AB), followed by a layer of cling wrap (GLAD, The Clorox Company of Canada LTD, Brampton, ON), Vetrap (3 M Animal Care Products, Maplewood, MN), Gorilla Tape, and lastly an additional layer of Vetrap to secure the catheter opening. The Gorilla Tape was used to restrict air entry and prevent moisture loss. This process was then repeated for the other hind foot.

#### Experimental infection and clinical evaluation

The experimental timeline is shown in Fig. 4. On day 0 calves were individually inoculated starting with the MI group followed by the IN group. Calves were moved from their pens to the manual chute by way of an alleyway. Calves were restrained, their hind foot lifted, tied with a rope, then the outermost layer of the Vetrap removed. Depending on the group, either the growth media (MI) or inoculum (IN) was deposited through the catheter using the prepared syringe for the individual calf. The syringe was then removed, and the foot re-wrapped with Vetrap to cover the opening of the catheter. This process was then repeated for the other hind foot.

Calves were monitored daily for wrap integrity, signs of discomfort due to wrapping, and clinical signs of lesion development (lameness). Lameness was defined as altered gait or reluctance to bear weight. Wrap-associated lameness was assigned to calves that presented lameness when no DD lesion was present. On days 13 and 20, calves were again taken to the chute where all wrapped feet were re-moistened with 10 ml of growth media using the same procedure followed at inoculation. At this time wrap integrity was checked and reinforced where needed with Gorilla Tape. On day 28 all existing wraps were removed, both hind feet clinically appraised for DD, MNT (ProdPlus, Topcat Metrology Ltd, Ely, Cambridgeshire, England) measured, and feet with DD, biopsied using a 4 mm punch, treated with topical oxytetracycline (5 g, Dominion, Veterinary Laboratories LTD, Winnipeg, MB), and if needed Metacam (6.5 ml subcutaneously, Boehringer Ingelheim, Burlington, ON) was administered for pain relief. Calves were monitored for 3 weeks post treatment for healing (no sign of pre-existing lesion) and return to normal skin as visually appraised.

Calves that exhibited clinical signs of lesion development (lameness) prior to completing the 28-day protocol were assessed immediately for the presence of DD. Calves were restrained in the manual chute, foot wrap from the lame limb removed and foot clinically appraised for DD. If DD was not present the foot was remoistened with growth media and rewrapped. If DD was present, the wrap was not re-applied, and the non-lame foot assessed for presence of DD. This was the experimental endpoint for these calves, and they were processed in accordance with the protocol for day 28 of the experiment.

### Quantitative real-time polymerase chain reaction (qPCR)

Biopsies from donor beef heifers and IN-DD feet were processed in accordance with published methods [22, 40]. Briefly, deoxyribonucleic acid (DNA) from biopsy samples were purified with the Qiagen DNeasy blood and tissue kit (Qiagen, Hilden, Germany) according to manufacturer’s recommendations, then normalized to 10 ng/µl using Qubit dsDNA HS kit (Life Technologies, Carlsbad, CA). Bacteria associated with DD lesions were determined by qPCR absolute quantification (CFX96 real-time system; Bio-Rad Laboratories Inc., Hercules, CA) using three qPCR assays developed to target three different *Treponema* spp. [40], *F. necrophorum *[41], *P. levii*, *Fusobacterium* sp., and *B. pyogenes* [22].

Standards for the Treponema qPCR were pre- pared as plasmid copy numbers as described by Beninger et al. [40] and genomic DNAs purified from DD isolates were used for the other qPCR assays, as described by Caddey et al. [22]. Prior to each reaction, standards for all three assays were measured with the Qubit dsDNA HS kit. The qPCR conditions were initial denaturation at 95 °C for 5 min, followed by 35 cycles of 95 °C for 30 s, 58 °C for 30 s, and 72 °C for 90 s and an additional step at 72 °C for 10 min. The required amplification efficiencies for all qPCRs ranged from 90 to 110% and all no-template controls (NTC) were negative. Additionally, for all qPCR assay’s, DNA extraction controls were tested and were all negative. Absolute abundances were then normalized by biopsy tissue weight allowing for comparison of bacterial quantities based on copy numbers per milligram of biopsy tissue.

### Behaviour and pain

All pens were equipped with the Lorex 4 k Ultra HD cameras (Lorex Technology Inc., Markham, ON) for continuous recording of lying and standing behaviour. Lying was defined as lying or resting with the whole body on the floor in any posture. Standing was defined as all four legs over the floor in any posture.

An ear accelerometer (CowManager system, Agis, Harmelen, The Netherlands) attached to the radio-frequency identification (RFID) tag (Allflex, Dallas, TX), positioned in the proximal half of the right ear was used to measure behaviour (day -2; Fig. 4). The CowManager system is a 3-dimensional ear accelerometer that measures ear movements and quantifies rumination, feeding, high activity, activity, and inactivity time through a proprietary algorithm. Data was collected by the ear sensor minutely and averaged on an hourly basis for the duration of the experiment.

An algometer (ProdPlus, Topcat Metrology Ltd, Ely, Cambridgeshire, England) was used to quantify pain at the lesion site on both hind feet. The algometer was fitted with a 1 mm brass tip which was pressed against each lesion site at a rate of 2 N/sec. The amount of pressure, measured in Newtons of force, required to elicit a withdrawal response (withdrawal of the foot) was recorded as the MNT.

### Data Management and Statistical analyses

Data from the videos captured by the Lorex cameras system was extracted using the BORIS software [42]. Using a 5-min instantaneous sampling interval, proportion of time spent lying and standing per day were summarized.

The CowManager system generated Microsoft Excel spreadsheets with all the behaviour (rumination, feeding, high activity, activity, and inactivity) measured throughout the study. Hourly data for each behaviour was converted to percent time per day ((daily minutes / total minutes) * 100). High activity and activity were merged and categorized as activity.

Average MNT was calculated by averaging the MNT measurements obtained at the four abrasion sites.

Descriptive statistics were calculated to summarize behaviour using IBM SPSS Statistics for Windows, version 26 (IBM Corp., Armonk, N.Y., USA). The experimental unit was animal.

## Data Availability

The datasets used and/or analysed during the current study are available from the corresponding author on reasonable request.

## Abbreviations

*B. pyogenes*: *Bacteroides pyogenes*
DC: Dewclaws
DD: Digital dermatitis
DNA: Deoxyribonucleic acid
*F. necrophorum*: *Fusobacterium necrophorum*
*Fusobacterium* sp.: *Fusobacterium* species
HB: Heel bulbs
ID: Identification
IN: Inoculated
IN-DD: Inoculated diseased
IN-NDD: Inoculated not diseased
LH: Left hind
MI: Mock-inoculated
MNT: Mechanical nociceptive threshold
M-stage: Mortellaro stage
NTC: No-template controls
qPCR: Quantitative polymerase chain reaction
*P. levii*: *Porphyromonas levii*
RFID: Radio-frequency identification
RH: Right hind
*T. medium*: *Treponema medium*
*T. pedis*: *Treponema pedis*
*T. phagedenis*: *Treponema phagedenis*
